# Pilot Study on the Use of Low-Field Nuclear Magnetic Resonance as a Noninvasive Tool for Monitoring Mucus in Obstructive Lung Diseases

**DOI:** 10.3390/ijms27146355

**Published:** 2026-07-17

**Authors:** Alice Biasin, Gianmarco Sarro, Paola Confalonieri, Francesco Salton, Marco Confalonieri, Alessandra Abriani, Giuseppina Campisciano, Manola Comar, Domenico Tierno, Federica Tonon, Alessandra Adrover, Claudia Venditti, Gabriele Grassi, Mario Grassi, Michela Abrami

**Affiliations:** 1 Department of Medicine, Surgery and Health Sciences, University of Trieste, Strada di Fiume 447, 34149 Trieste, Italy; alice.biasin@units.it (A.B.); gianmarco.sarro@studenti.units.it (G.S.); mcomar@units.it (M.C.); domenico.tierno@units.it (D.T.); federica.tonon@asugi.sanita.fvg.it (F.T.); 2 Pulmonology Department, Cattinara University Hospital, Strada di Fiume 447, 34149 Trieste, Italy; paola.confalonieri@asugi.sanita.fvg.it (P.C.); francesco.salton@asugi.sanita.fvg.it (F.S.); mconfalonieri@units.it (M.C.); alessandra.abriani@studenti.units.it (A.A.); 3 Institute for Maternal and Child Health-IRCCS Burlo Garofolo, 65/1 Via Dell’istria, 34137 Trieste, Italy; giuseppina.campisciano@studenti.units.it; 4 Department of Chemical Engineering, Materials, Environment, Sapienza University of Rome, Via Eudossiana 18, 00184 Rome, Italy; alessandra.adrover@uniroma1.it (A.A.); claudia.venditti@uniroma1.it (C.V.); 5 Department of Engineering and Architecture, University of Trieste, Via Valerio 6/A, 34127 Trieste, Italy; michela.abrami@dia.units.it

**Keywords:** low-field NMR, obstructive lung disease, monitoring, microbiome, disease index, *FEV*_1_/TNFα/IL-6

## Abstract

Patients with muco-obstructive lung disease (MOLD) exhibit chronic bronchitis and inflammation, along with a progressive decline in lung function. Lung monitoring is typically performed using spirometry, especially by measuring the forced expired volume in the first second (*FEV*_1_). However, the limitations of spirometry motivated the exploration of alternative approaches. The spin–spin relaxation time (*T*_2*m*_) and the spin–lattice relaxation time (*T*_1*m*_) of sputum water hydrogens were measured using low-field nuclear magnetic resonance (LF-NMR) in 38 MOLD patients and 16 controls. The levels of TNFα/IL-6, the sputum microbiome composition/amount/indices and *FEV*_1_ were determined in parallel. We also investigated the correlation between *T*_2*m*_/*T*_1*m*_ and the disease index (*I_D_*); *I_D_*, calculated relying on patient *FEV*_1_/TNFα/IL-6/bacteria concentration *C_b_* values, is a measure of the patient’s distance from the average healthy control. We observed the following significant correlations: *T*_2*m*_/*T*_1*m*_ with *I_D_*, *T*_2*m*_ with *C_b_*, a potential correlation of *T*_2*m*_ with the bacteria genera Streptococcus and Staphylococcus, *T*_2*m*_ with the Shannon index, which reflects the broadness of the bacterial community in the sputum, and *T*_2*m*_ with TNFα. *FEV*_1_ did not show any correlation. Our noninvasive/radiation-free/portable method of *T*_2*m*_/*T*_1*m*_ measurement shows potential value in monitoring lung conditions in MOLD patients and may contribute to improved clinical decision-making.

## 1. Introduction

Chronic obstructive pulmonary disease (COPD) and bronchiectasis are common chronic respiratory diseases [1]. COPD is a heterogeneous condition, with considerable variation observed between individuals in terms of clinical manifestations and pathophysiological features [2]. Its main characteristics include persistent symptoms and impaired lung function as a consequence of airway inflammation, small airway obliteration, and alveolar destruction [1]. Non-cystic fibrosis bronchiectasis (NCFB) is a respiratory disease characterized by the permanent enlargement of parts of the lung’s airways, leading to mucus accumulation and increased susceptibility to infection. Typically, patients develop acute lung infections associated with fever, dyspnea, and excessive mucus production [3].

Although COPD and NCFB are clinically distinct entities, both fall within the group of “muco-obstructive lung diseases” (MOLD), in which mucus production and its properties play a crucial role in determining disease progression [4]. Both diseases are associated with an accelerated loss of lung function [4], secondary to bacterial infections that trigger inflammation [5]. The frequency of exacerbations necessitates intensification of outpatient treatment and correlates directly with an unfavorable prognosis [6,7]. Exacerbations occur in approximately 20% of patients within the first 30 days following hospital discharge, 30% within the first 3 months, and 50% within 6 months [8].

Patient monitoring is routinely based on lung function tests such as spirometry, which includes measurement of the forced expiratory volume in the first second (*FEV*_1_). However, spirometry is not currently recommended for assessing the severity of MOLD exacerbations. Exacerbated patients often exhibit muscle weakness, bronchospasm, and air trapping, which make spirometry difficult to perform accurately [8,9]. Moreover, *FEV*_1_ is insensitive to early structural changes, depends on effort, age, height, and sex, and cannot assess mucus properties. Finally, the severity of obstruction assessed by *FEV*_1_ does not directly reflect the overall disease burden or systemic manifestations in individual patients [10,11].

Here, we propose the use of low-field nuclear magnetic resonance (LF-NMR) to measure the spin–spin (*T*_2*m*_) and spin–lattice (*T*_1*m*_) relaxation times of water protons in sputum obtained by spontaneous expectoration from MOLD patients. Our previous results indicate that LF-NMR can effectively monitor lung conditions in patients with cystic fibrosis (CF) [12]. In particular, we demonstrated an inverse correlation between *T*_2*m*_ and sputum structural characteristics altered by the pathological increase in substances such as DNA, mucins, and bacteria [12]. Furthermore, we reported an inverse correlation between *T*_2*m*_ and the inflammatory biomarkers tumor necrosis factor alpha (TNFα) and interleukin-1β (IL-1β), as well as a direct correlation with *FEV*_1_ [12,13]. Notably, sputum contains similar lung mucus constituents along with cells, such as neutrophils and macrophages, DNA, microorganisms, and inflammatory mediators [14]. Thus, sputum is widely regarded as a “window into the lower respiratory tract”, as it provides direct insight into the presence of infection, the extent of inflammation, and the nature of immune responses occurring within the pulmonary environment. Furthermore, sputum constitutes a noninvasive and readily repeatable biological specimen, in contrast to more invasive diagnostic procedures such as bronchoscopy or bronchoalveolar lavage, which require specialized equipment and more complex clinical organization.

In this study, we extend this novel monitoring approach to MOLD patients. In 38 MOLD sputum samples and 16 healthy controls, we measured *T*_2*m*_ and *T*_1*m*_ alongside levels of the pro-inflammatory cytokines tumor necrosis factor alpha (TNFα) and interleukin-6 (IL-6), which are elevated in COPD patients [15,16]. Additionally, we explored the relationship between *T*_2*m*_/*T*_1*m*_ and *FEV*_1_. Given the strong link between the respiratory tract microbiome and MOLD exacerbations [17], we also characterized the microbiome composition of the sputum samples. Notably, bacterial communities in the lungs have been shown to display a composition similar to that of the upper airways [6]. Finally, we analyzed correlations between *T*_2*m*_/*T*_1*m*_ and various biological parameters in MOLD patients, including those under acute exacerbation (22 patients) and those in stable conditions (16 patients).

## 2. Results

To explore the value of *T*_2*m*_/*T*_1*m*_ measured in the sputum (see details on their evaluation in Section 4.4 and Section 4.5) in monitoring lung functions in MOLD patients, *T*_2*m*_/*T*_1*m*_ were compared with *FEV*_1_, with the inflammatory cytokines TNFα/IL-6 and with bacterial metrics (bacterial concentration *C_b_*, Shannon index *Sh*, Equitability index *Eq*), variables commonly analyzed in sputum samples to evaluate airway inflammation in MOLD [15,16,17].

### 2.1. Validation of the Selected Parameters

We first proved the ability of the considered parameters to distinguish between patients and healthy controls, as reported in Table 1a and Table 1b, respectively.

**Figure 1:**
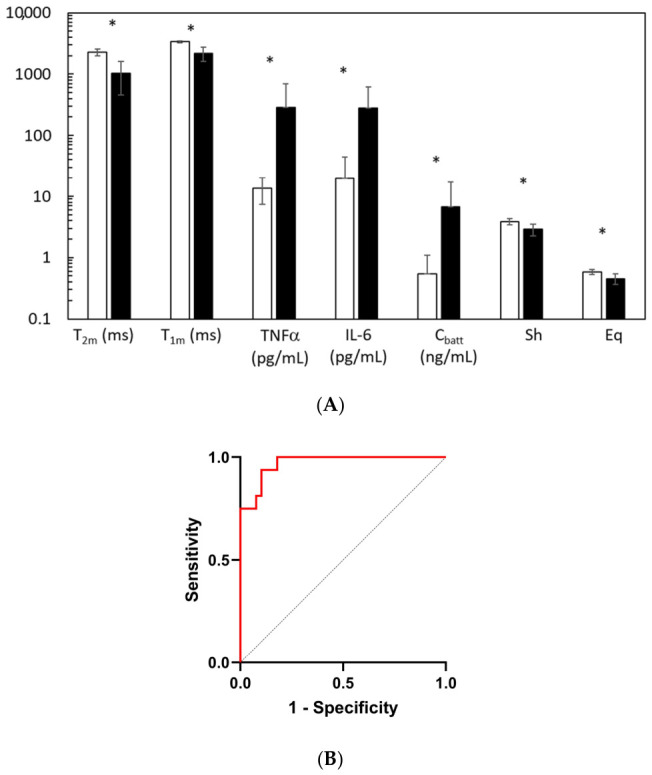
(**A**) Comparison among the average parameters from healthy (white) and MOLD (black) samples. From left to right, the data represent the average spin–spin relaxation time (*T*_2*m*_), the average spin–lattice relaxation time (*T*_1*m*_), concentrations of TNFα and IL-6, DNA bacterial concentration (*C_b_*), the Shannon (*Sh*) and Equitability (*Eq*) indices. Test t: t*T*_2*m*_ (*p* = 0.05, 53) < 8; t*T*_1*m*_ (*p* = 0.05, 53) < 4.12; tTNFα (*p* = 0.05, 53) < 2.7; tIL-6 (*p* = 0.05, 53) < 3.29; t*C_b_* (*p* = 0.05, 53) < 2.46; t*Sh* (*p* = 0.05, 53) < 5.59; t*Eq* (*p* = 0.05, 53) < 5.56. Data are reported as mean ± SD. (**B**) Area under the ROC curve for T_2m_: 0.971 (CI 0.935–1.000) *p*-value < 0.0001 cut-off > 1881 ms (Sens: 0.937 Spec: 0.897). * *p* < 0.05.

Figure 1A presents the average values from healthy samples (white) and MOLD patients (black): statistically significant differences were observed between each pair of healthy and diseased samples. The fact that *T*_2*m*_ is lower in MOLD compared with healthy sputum samples confirms our previous observation in CF patients [12]; moreover, a receiver operating characteristic (ROC) analysis (Figure 1B) indicates a high accuracy of *T*_2*m*_ in distinguishing patients from healthy controls. We also show that *T*_1*m*_ is statistically lower in the sputum of affected patients compared to healthy subjects. Thus, these data prove the validity and significance of *T*_2*m*_/*T*_1*m*_ evaluation in MOLD patients.

As expected, our data also show that TNFα, IL-6, and *C_b_* are lower in healthy subjects compared to MOLD. Finally, it is interesting to note that *Sh*/*Eq*, typically close to their maximum (4 and 1, respectively) in healthy subjects, are significantly reduced in MOLD patients. High *Sh* and *Eq* values reflect the biodiversity richness, indicating that no single bacterial taxa is dominant in the sputum; this is a condition typical of healthy subjects. In contrast, low *Sh*/*Eq* values indicate a pathological condition characterized by the prevalence of one bacterial taxon responsible for exacerbation.

### 2.2. Correlation Among T_2m_/T_1m_ and I_D_

**Figure 2:**
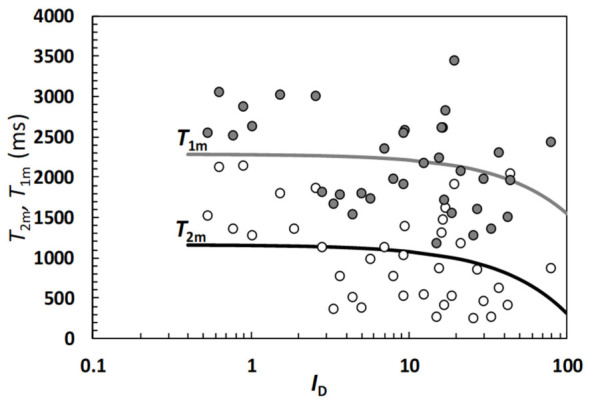
Dependence of the average relaxation times *T*_2*m*_ (white circles) and *T*_1*m*_ (gray circles) on the disease index, *I_D_*. A significant inverse correlation between *I_D_* and *T*_2*m*_/*T*_1*m*_ exists: T_2m_ − r_sp_= −0.37, *p* < 0.023; T_1m_ − r_sp_ = −0.34, *p* < 0.038). Continuous lines indicate the existing linear correlation.

To easily and synthetically describe lung conditions, we propose a “disease index” (*I_D_*), representing the patient’s distance from the average control. I_D_ was calculated on the basis of the considered patient biomarkers (FEV_1_/TNFα/IL-6/bacteria concentration *C*_b_) and on the average values of the same markers referring to controls (Section 4.10). *I_D_* is equal to zero for the average healthy subject (control) and progressively increases with the worsening of the lung conditions. Our data shows a significant inverse linear correlation between *I_D_* and *T*_2*m*_/*T*_1*m*_ (Figure 2).

Additionally, *T*_2*m*_/*T*_1*m*_ correlate each other (r_sp_ = 0.836, *p* < 10^−4^; *T*_1*m*_ = (0.81 ± 0.087) × *T*_2*m*_ + (1337 ± 103)). As *I_D_* does not mathematically depend on *T*_2*m*_/*T*_1*m*_ (see Equation (20) in M&M Section 4.10), the existence of these correlations supports the use of *T*_2*m*_/*T*_1*m*_ as parameters to monitor lung condition. Notably, we also found significant statistical correlations among *I_D_* and *Sh* (r_sp_ = −0.44, *p* < 0.0048) and *I*_D_ with *Eq* (r_sp_ = −0.4, *p* < 0.001). Thus, *T*_2*m*_/*T*_1*m*_ have the power to reflect and represent the multiple parameters considered to monitor lung conditions.

**Figure 3:**
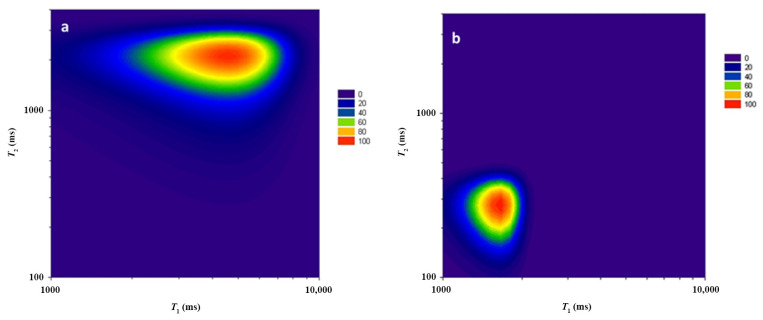
*T*_2_–*T*_1_ intensity plane for individual patients. The y-axis represents *T*_2_ relaxation times, while the x-axis represents *T*_1_ relaxation times. Color scale indicates signal intensity from violet (low) to red (high). (**a**) Patient with good lung condition (PN20, *I_D_* = 2.8) shows a broad distribution with higher intensity at longer relaxation times. (**b**) Patient with poor lung condition (PN27, *I_D_* = 47.8) exhibits a narrow distribution with maximum intensity at shorter relaxation times.

For an easy and rapid use of *T*_2*m*_/*T*_1*m*_ in the clinic, it is possible to visualize them through an image in the *T*_2_/*T*_1_ plane (details in M&M Section 4.6, Section 4.7 and Section 4.8), as reported in Figure 3.

As an example, we consider two patients, either with good (Figure 3a, PN20 of Table 1a, low *I_D_* = 2.8) or poor (Figure 3b, PN27 of Table 1a, high *I_D_* = 47.8) lung conditions, as defined by the *I_D_* value. The colored peak indicates the product of the intensities (*A*_2*i*_/*A*_1*i*_), associated, respectively, with *T*_2_/*T*_1_. When the red peak appears in the upper right zone of the *T*_2_/*T*_1_ plane (Figure 3a), *T*_2*m*_/*T*_1*m*_ have high values reflecting good lung conditions (patient PN 20, *I_D_* = 2.8, *T*_2*m*_ = 1798 ms, *T*_1*m*_ = 3017 ms). In contrast, when the red peak appears in the lower left zone of the *T*_2_/*T*_1_ plane (Figure 3b), the lung conditions are poor (patient PN 27, *I_D_* = 47.8, *T*_2*m*_ = 257 ms, *T*_1*m*_ = 1348 ms). Thus, this representation of *T*_2*m*_/*T*_1*m*_ can be easily and rapidly interpreted by a clinician.

### 2.3. Correlation Among T_2m_ and FEV_1_/TNFα/IL-6/C_b_

As *T*_2*m*_ correlates with *T*_1*m*_, and *T*_2*m*_ determination is faster than *T*_1*m*_, from now on, we focus on *T*_2*m*_ only. Our data (Table 2) show a significant indirect correlation of *T*_2*m*_ with TNFα and *C_b_*, which reflect the local lung inflammation and the DNA bacterial load, respectively. Additionally, TNFα and *C_b_* exhibit a direct correlation, as well as TNFα with IL-6. Notably, *FEV*_1_ does not show any significant correlation with any of the parameters considered.

### 2.4. Correlation Among T_2m_ and the Bacterial Parameters d_M_, $P_{MAX}^{\%}$ D_0_, S_h_ and E_q_

To investigate the correlation of *T*_2*m*_ with the infecting bacterial Amplicon Sequence Variants (ASVs), we considered the statistical indices (*d*_M_, $P_{MAX}^{\%}$ *D*_0_) and *Sh*/*Eq* as defined in M&M Section 4.9. These indices define the broadness of the bacterial community, i.e., how much the percentage of each ASV deviates from the mean value. While *Sh* and *E*q represent the classical Shannon and Equitability indices, *d*_M_ and *D*_0_ are two novel indices, mathematically defined by Equation (17) and Equation (19), respectively, of M&M Section 4.9, allowing to characterize the microbiome community distribution. In particular, *d*_M_ measures the community distance from its average component (bacterial ASVs) characterized by: (1) a zero distance from the average component (itself) and (2) the average percentage. Notably, this parameter allows a simple and immediate visualization of the microbiome distribution as depicted in Supplementary Information Figure S1. On the other hand, *D*_0_ represents a strategy of measuring the community distance from a reference condition represented by the ideal community characterized by *d*_M_ = 0 and a vanishing value of the maximum percentage ($P_{MAX}^{\%}$ = 0). While in healthy conditions, many equally represented different bacterial ASVs coexist in the sputum (*d*_M_ ≈ $P_{MAX}^{\%}$ ≈ *D*_0_ ≈ 0), in pathological conditions, so some pathogenic bacteria increase [18]. This leads to the prevalence of a limited number of bacterial types so that both *d*_M_ and *D*_0_ increase (Figure S1). The correlations were evaluated at the bacterial ASVs level (Table 3).

*T*_2*m*_ correlates directly with *Sh*, suggesting that *T*_2*m*_ can reflect, at least in part, the prevalence of one bacterial ASV responsible for disease exacerbation. To the best of our knowledge, this is the first demonstration that parameters obtained from LF-NMR can correlate with bacterial prevalence in a biological sample.

Table 3 also shows a significant correlation among *d*_M_, $P_{MAX}^{\%}$*D*_0_, *Sh*, *Eq* and both IL-6 and *C_b_*. The direct correlation of *C_b_* with *d*_M_, $P_{MAX}^{\%}$ and *D*_0_ indicates that the increase in the DNA bacteria concentration relates to the narrowing of the bacteria distribution. The existence of an inverse correlation between *C_b_* and *Sh*/*Eq* indicates the growth of a predominant bacterial ASV. Notably, TNFα does not correlate with *d*_M_, $P_{MAX}^{\%}$ and *D*_0_, *Sh*/*Eq*, indicating that the amount of this inflammatory cytokine is quantitatively independent from bacterial composition. In contrast, the levels of IL-6 directly correlate with *d*_M_, $P_{MAX}^{\%}$ and *D*_0_ and indirectly with *Sh*/*Eq*. *FEV*_1_ does not show any significant correlation (Table 3).

Finally, we found a significant correlation among the microbiome indices (*d*_M_, $P_{MAX}^{\%}$*D*_0_, *Sh*, *Eq*) evaluated at the bacterial phylum (≈10 individuals) and at the bacterial genus (≈1000 individuals) levels (*d*_M_ − r_p_ = 0.56, *p* < 2 × 10^−4^; $P_{MAX}^{\%}$ − r_p_ = 0.55, *p* < 4.3 × 10^−4^; *D*_0_ − r_p_ = 0.57, *p* < 2 × 10^−4^; *Sh* − r_p_ = 0.74, *p* < 10^−4^, *Eq* − r_p_ = 0.62, *p* < 10^−4^).

### 2.5. Correlation Among T_2m_ and the Bacterial Phyla/Genus

Previous studies have shown that in MOLD patients, the changes in sputum microflora were related to disease progression, frequency of exacerbations, level of inflammation and treatment [19]. Thus, we analyzed the sputum microflora in our samples to study the correlation with the parameters we selected.

**Figure 4:**
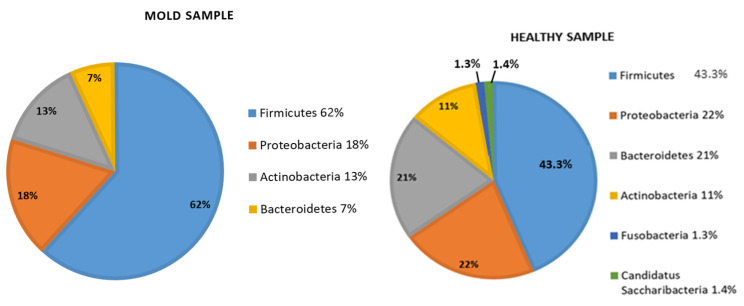
Relative abundance of bacterial phyla (>1%) in healthy and MOLD samples. Colors correspond to phyla as indicated in the legend, where percentage values are reported for each phylum. Healthy samples show Firmicutes (43.3%), Proteobacteria (22%), Bacteroidetes (21%), Actinobacteria (11%), Fusobacteria (1.3%), and Candidatus Saccharibacteria (1.4%). MOLD samples show Firmicutes (62%), Proteobacteria (18%), Actinobacteria (13%), and Bacteroidetes (7%).

At the phylum level, our data (Figure 4) indicate that Firmicutes, Actinobacteria, and Proteobacteria represent the most frequent phyla in both patients and healthy subjects; however, in healthy subjects, Firmicutes concentration is much lower, being the 41.3% of total *C_b_*, while in the MOLD group, it is the 61.3% of total *C_b_*, in line with the literature [6,20].

At the bacterial genus level, the most common bacteria are *Streptococcus* (31.9%), *Rothia* (10.51%), *Haemophilus* (3.1%), *Staphylococcus* (3.1%) and *Pseudomonas* (2.6%). In healthy sputum, the most abundant are *Streptococcus* (19.0%), *Prevotella* (9.3%), *Neisseria* (6.9%), *Veillonella* (4.7%) and *Granulicatella* (4.6%). This agrees with Taherkani [21], who indicated that *Veillonella* and *Prevotella* are the high-frequency constituting genera in healthy lungs. Ramshesh’s findings [20] also evidenced that *Prevotella* abundance is greater in healthy individuals and is significantly associated with better lung function and reduced symptoms in COPD patients.

Starting from the above data, we searched for possible correlations of *T*_2*m*_/TNFα/*Sh*/*FEV*_1_ with the concentration of the different bacterial genera. For this purpose, we selected 42 bacterial genera characterized by the highest concentration (typically, at the genus level, we have ≈100 different bacteria) as they represent more than 90% of all bacteria present in each sample. Table 4 shows that *T*_2*m*_ and *Sh* correlate with the concentration of different bacterial genera that include *Streptococcus* and *Staphylococcus*, the most abundant genera in MOLD sputum [21]. The search for false positives according to the Benjamini–Hochberg test reveals that the correlation between *Sh* and *C_b_* also takes place when the false discovery rate (FDR) level is equal to 0.01 (Figure S2). In the case of *T*_2*m*_, instead, the percentage of correlation is bigger than zero only when the FDR level > 0.07 (Figure S2). These outcomes further stress the informative value of *T*_2*m*_ and show a potential correlation of *T*_2*m*_ with bacterial genera. No significant correlations were found for TNFα and *FEV*_1_.

### 2.6. MOLD Clustered in Acute Exacerbation (aEX) and Stable (ST)

We then explored the correlation of *T*_2*m*_ with the parameters considered in this work in patients subdivided into “acute exacerbation” (aEx–22 patients) or “stable condition” (ST–16 patients). In aEX (Table 5), but not in the ST group, a significant correlation of *T*_2*m*_ with TNFα and *C_b_* takes place.

In line with this, a statistically significant correlation occurs between *C_b_* and TNFα. Thus, the above findings suggest that *T*_2*m*_ associates with the exacerbation in MOLD patients. It is conceivable that *T*_2*m*_ may reflect a significant mucus thickening secondary to the increase in bacteria concentration, inflammatory cytokines and other pathological substances.

We also observed that IL-6, but not TNFα/*T*_2*m*_/*FEV*_1_, displays (Table S1) a statistically significant correlation with the indices describing the microbiome distribution in the aEX group. Finally, we found that aEX group is characterized by an increase in genera usually responsible for infective exacerbations [22,23] such as *Lactobacilli*, *Staphylococcus* and *Pseudomonas*, which are almost absent in ST (Figure S3).

## 3. Discussion

Identifying parameters that can accurately assess the severity of lung disease in MOLD remains a significant challenge [8]. Here, we explore the ability of *T*_2*m*_/*T*_1*m*_ to monitor lung conditions in MOLD patients. In addition to showing that all the markers considered (*T*_2*m*_, *T*_1*m*_, TNFα, IL-6, *C_b_*, *Sh*, *Eq*) differ significantly between healthy subjects and MOLD patients (Figure 1), we defined a novel comprehensive clinical indicator, the disease index (*I_D_*). This index synthetically groups different parameters related to lung conditions, indicating how far a patient’s lung status deviates from normality. The proposed *I_D_* remains to be externally validated and the present findings should be confirmed in larger independent cohorts. We observed that *T*_2*m*_/*T*_1*m*_ inversely correlates with *I_D_* (Figure 2), in agreement with what we previously reported in CF patients for *T*_2*m*_ [12,24]. Notably, *T*_2*m*_/*T*_1*m*_ can be graphically represented (Figure 3), enabling easy interpretation by clinicians and facilitating timely decision-making. Moreover, *T*_2*m*_/*T*_1*m*_ measurements do not cause discomfort to patients, as they only require expectoration. In contrast, FEV_1_ is highly technique- and effort-dependent, and some patients may find it uncomfortable.

By investigating the correlations between *T*_2*m*_ and *FEV*_1_, TNFα, IL-6, and *C_b_*, we found that *T*_2*m*_ correlates with *C_b_* (Table 2). This observation is consistent with our previous in vitro findings [12,25,26]. It should be noted that *C_b_* reflects total extracted DNA from sputum and may include DNA from non-viable bacteria as well as residual host DNA or contaminants; therefore, it may not precisely represent the viable bacterial load.

*T*_2*m*_ also inversely correlates with TNFα (Table 2), confirming our observations in CF patients [13]. This finding supports the value of *T*_2*m*_ as a general marker for monitoring productive lung diseases. The inverse relationship between *T*_2*m*_ and TNFα suggests that TNFα is linked to mucus solid content and nanostructure, two factors that strongly influence *T*_2*m*_*,* as well documented in [27,28,29,30]. Indeed, during inflammation, TNFα is released by bronchial epithelial cells and stimulates mucin secretion in the airway epithelium [31,32], increasing mucus viscosity and, consequently, reducing *T*_2*m*_. Notably, no significant correlation was found between *T*_2*m*_ and IL-6, which is commonly used to monitor COPD progression [15,33]. This suggests that IL-6 levels may not be directly related to mucus solid content or nanostructure in our cohort. However, we cannot exclude the possibility that the sample size was insufficient to detect a significant relationship, and further investigation is required. Despite this, our data (Table 3) indicate that IL-6 correlates with the distribution of the bacterial community, as determined by indices such as *d_M_*, $P_{MAX}^{\%}$, *D*_0_ and *Sh*/*Eq*. These indices reflect how much the relative abundance of each bacterial ASV deviates from the mean. In diseased patients, they typically indicate the dominance of a limited number of bacterial species responsible for lung infection.

FEV_1_ does not correlate with TNFα, IL-6, or *C_b_* (Table 2), nor with bacterial indices such as *d_M_*, $P_{MAX}^{\%}$, *D*_0_ and *Sh*/*Eq* (Table 3). Therefore, using *FEV*_1_ alone to monitor lung conditions in MOLD patients provides limited information. In contrast, the combined use of *FEV*_1_ and *T*_2*m*_ may lead to a more comprehensive understanding of lung disease.

Our data (Table 4) also show that T_2m_ correlates with the abundance of bacterial genera such as *Streptococcus* and *Staphylococcus*, which are often involved in MOLD-related lung disease [21]. It should be noted that whereas T_2m_-genus correlations do not meet a very stringent false discovery rate threshold (q = 0.05), this does not preclude the presence of meaningful associations. The progressive emergence of signals at slightly more permissive thresholds (q > 0.07) instead suggests a distributed pattern of effects. Increasing the number of samples (work in progress) may strengthen the correlation strength. The potential correlation with *Staphylococcus* is particularly noteworthy, as this genus is known to produce biofilms [34] containing proteases and surfactant-like proteins [35]. Additionally, *Staphylococcus* synthesizes surface-anchoring proteins that bind multiple ligands on the lung epithelium, facilitating bacterial adherence. Together, these factors contribute to alterations in mucus solid content and nanostructure, increasing viscosity, and correlate with *T*_2*m*_. Notably, *FEV*_1_ does not correlate with any relevant bacterial genera, further highlighting its limitations.

Interestingly, in patients with acute exacerbations (aEX), but not in stable (ST) patients, *T*_2*m*_. shows significant correlations with TNFα and *C_b_* (Table 5), while IL-6 correlates with microbiome indices (Table S1). Furthermore, genera such as *Lactobacillus*, *Staphylococcus*, and *Pseudomonas* were observed only in the aEX group, whereas *Prevotella* was more prevalent in stable and healthy individuals. These findings support the concept that *T*_2*m*_. could become a valuable tool for monitoring disease progression and guiding clinical decisions, potentially improving treatment timeliness. However, longitudinal studies assessing *T*_2*m*_. dynamics over time are required for full validation.

Finally, although not shown graphically in Section 2, our data suggest that *T*_2*m*_. values do not differ substantially between COPD and bronchiectasis patients. However, the microbiome profile and its relationship with *T*_2*m*_. appear to be disease-specific, differing between these conditions. We plan to investigate this aspect further using a larger, multicenter cohort (work in progress).

This study has several limitations that should be considered when interpreting the results. First, the sample size is relatively small. Second, this was a single-center study, and thus its findings may not be fully representative of the broader MOLD population; confirmation in multicenter studies is needed. Third, although no patients were receiving mannitol, hypertonic saline, or nebulized saline at the time of exacerbation or sputum collection—and all were on comparable long-term bronchodilator regimens—detailed information on mucolytic therapy (including duration, dosage, and adherence) was not consistently available. This limits our ability to fully assess the potential influence of mucolytics on sputum characteristics. Finally, it should be considered that T_2m_ is influenced by multiple physicochemical factors, not exclusively by solid concentration and microstructure we considered here (see Figure S4). Thus, the technique has a certain degree of approximation. Despite this, it is used to evaluate biofluids and soft biological tissues, providing noninvasive assessments of hydration, viscosity, and macromolecular content [36].

In conclusion, we demonstrate that in MOLD patients, *T*_2*m*_. correlates with traditional biomarkers (TNFα, *C_b_*, and *Sh*) as well as with the proposed disease index (ID). We also introduce a method for simultaneous visualization of *T*_2*m*_. and *T*_1*m*_., enabling fast and intuitive interpretation, even by non-specialized personnel. In contrast, *FEV*_1_ does not correlate with disease indices or key bacterial genera. Taken together, our findings—combined with the noninvasive, radiation-free, portable, and easy-to-measure nature of *T*_2*m*_.—suggest that it can effectively complement *FEV*_1_ in monitoring lung conditions in MOLD patients and improve clinical decision-making and treatment timing.

## 4. Materials and Methods

### 4.1. Samples Collection

Spontaneously expectorated sputum samples (1–2 mL) were obtained from 38 consecutive patients with heterogeneous clinical conditions (Table 1), following a protocol approved by the Ethics Committee of the University of Trieste (approval no. 118, 2 December 2021). All procedures were conducted in accordance with the Committee’s guidelines and regulations, and written informed consent was obtained from each participant. Only patients who were able to expectorate provided samples. As previously reported in cystic fibrosis patients [24], sputum was analyzed in its native form without using sputum induction techniques for two main reasons: (i) to establish a simple test suitable for non-specialized operators in routine clinical settings, and (ii) because sputum induction alters saline composition, thereby affecting *T*_2*m*_ measurements [12].

After collection, samples were gently mixed without further manipulation to preserve their structural properties, which could influence *T*_2*m*_ determination. From an average total volume of 2–3 mL, 1 mL was used for *T*_2*m*_ measurement. This volume is consistent with those reported in studies investigating cystic fibrosis sputum properties [37,38] and is considered representative of the whole sample. Immediately after collection, samples were divided into aliquots for different analyses (*T*_2*m*_/*T*_1*m*_ measurements, cytokine quantification, and microbiological analysis) and stored at −80 °C until processing. *T*_2*m*_/*T*_1*m*_ values were calculated as the mean of nine independent measurements. Previous studies in cystic fibrosis patients [13] showed that the coefficient of variation (CV = 2SD/mean × 100) for *T*_2*m*_ determination is approximately 2.5%. *T*_2*m*_ analysis required less than 1 min per sample, while the combined *T*_2*m*_/*T*_1*m*_ assessment required approximately 15 min. The procedure is straightforward and involves pipetting the sample into an LF-NMR tube.

Sputum samples were collected on the first day of hospitalization, prior to the administration of any antimicrobial therapy, as part of the standard diagnostic workflow. When clinically indicated, empirical antibiotic treatment was initiated after sample collection and completion of baseline assessments, typically within 24 h of admission.

COPD diagnosis was established according to GOLD guidelines [39], based on chronic respiratory symptoms and a post-bronchodilator *FEV*_1_*/FVC* ratio < 0.70. Disease severity was classified by GOLD stage according to post-bronchodilator *FEV*_1_ percentage of predicted values (GOLD 1: ≥80%; GOLD 2: 50–79%; GOLD 3: 30–49%; GOLD 4: <30%). Non-CF bronchiectasis was diagnosed based on clinical features (chronic cough, daily sputum production, and recurrent respiratory infections) together with radiological confirmation. All patients underwent high-resolution computed tomography (HRCT), and diagnosis required evidence of bronchial dilation according to accepted criteria (broncho-arterial ratio > 1, lack of bronchial tapering, and visualization of bronchi within 1 cm of the pleural surface). Spirometric data were interpreted using Global Lung Initiative (GLI-2012) reference equations adjusted for age, sex, height, and ethnicity.

To minimize contamination with food residues, patients refrained from eating for at least one hour prior to sampling. Samples were collected in sterile containers and immediately divided into three aliquots: one for *T*_2*m*_/*T*_1*m*_ analysis, one for cytokine measurement, and one for microbiome analysis. Monitoring tests were performed as part of routine day-hospital care. Exacerbation was defined as an acute worsening of dyspnea, cough, or sputum production within the previous 14 days, according to the 2023 GOLD guidelines [40].

A control group of 16 healthy subjects matched for age and sex was included. Following clinical examination and medical history assessment, none of the control subjects showed macroscopic evidence of chronic lung disease.

### 4.2. Bacterial DNA Identification

Bacterial DNA identification was performed by Labospace, Milan, Italy, according to the following protocol. Sputum samples were pre-treated with a 1 M NaOH solution at a 1:1 or 1:2 ratio according to the viscosity of the samples. After 15 min, the mixtures were vortexed and centrifuged at 13,000 rpm for 5 min. Bacterial DNA was extracted from the resulting pellets with the GRAM Bacteria Nucleic Acid Extraction Kit (TANBead, W61GS66, ACROBiosystems, Newark, DE, USA) following the manufacturer’s instructions on the Maelstrom 4810 TanBead automated extraction platform. The extracted DNA (3 µL for each sample) was used for microbiome analysis with the NGS method on the IonTorrent platform (Thermo Fisher Scientific, Waltham, MA, USA). For the library construction, the Ion 16S metagenomic kit was used, which analyses 7 of the 9 hypervariable regions of the bacterial 16S rRNA. Libraries were then sequenced on the Ion GeneStudio S5 Sequencer (Thermo Fisher Scientific, Waltham, MA, USA). Before the analysis, the sequences (or reads) obtained from sequencing were cleaned up using dedicated algorithms to remove short and low-quality reads. Sequences of less than 10 were not considered. The IonTorrent workflow generated high-quality read counts that, after filtering and removal of short/low-quality sequences, an average of 111,111.81 reads per sample (range: 16,952–237,397) were retained. The analysis of the raw sequence data was carried out by MicroBAT Software (version 2009), which performs the Taxonomic assignment of the individual reads by aligning with the RDP database (Ribosomal Database Project), version 11.4. Taxonomic assignment criteria were set as follows: minimum alignment length ≥100 bp, minimum percent identity ≥97%, minimum query coverage ≥80%, and E-value cutoff ≤1 × 10^−5^. Bacterial DNA identification was performed in a blinded fashion.

The total bacterial load (*C_b_*) was defined as the concentration of DNA obtained after extraction with the TANBead GRAM Bacteria kit. The extraction procedure includes selective binding and purification of bacterial DNA, with removal of most human DNA and host-derived contaminants. Nevertheless, C_b_ reflects total extracted DNA and may include DNA from non-viable bacteria and residual host/contaminants.

### 4.3. Cytokine Levels

The inflammatory cytokines TNFα and IL-6 were considered due to their involvement in COPD and NCFB patients [15]. The quantification was performed by Labospace, Milan, Italy, according to the following protocol. R&D System Human Magnetic Luminex Assay (Minneapolis, MN, USA), LXSAHM, was used to detect IL-6 and TNFα in sputum samples. The R&D LXSAHM used to quantify IL-6 and TNFα has a sensitivity of 0.8 pg/mL for IL-6 and 1.2 pg/mL for TNFα (as reported by the manufacturer). Values below the limit of detection (LOD) were treated as non-detectable and assigned as equal to half the LOD for statistical analyses, in accordance with common practice for cytokine quantification in sputum. Samples were diluted 2-fold with physiological water and centrifuged at 3000× *g* for 15 min due to the viscosity of the samples, then, with the kit diluent, they were no longer diluted. The standard and Microparticle cocktails and all other reagents were prepared following the manufacturer’s instructions. Sputum samples and standards were incubated with the microparticle cocktail for 2 h at room temperature on the shaker at 800 ± 50 rpm and then washed using a magnetic device designed to accommodate a microplate. Biotinylated Antibodies and Streptavidin-PE were then added following the manufacturer’s instructions. The plates were read with the Flex Map 3D analyzer (Luminex Corporation, Austin, TX, USA), and the results were reported in real time by Bio Plex 6.2 software. The reported data identify the analyte detected and the relative amount present in the sample, derived from the magnitude of the PE-derived signal. Cytokine level determination was performed in a blinded fashion.

### 4.4. Portable Low-Field NMR

LF-NMR relies on the ability of hydrogen atoms’ dipole to react to the perturbation of an external constant magnetic field *B*_0_ (conventionally directed along z axis, i.e., the longitudinal direction), where they are embedded. Basically, the application of a radio frequency pulse *B*_1_, lying in the x-y plane perpendicular to *B*_0_, induces dipole orientation in the x-y plane. After *B*_1_ removal, dipoles tend to return to their initial alignment with B_0_ (relaxation). The relaxation process can be described by two characteristic times. The average spin–spin relaxation time, *T*_2*m*_, which measures the extinction of *M_xy_*, the x-y (or transversal) component of the magnetization vector (*M*), and the average spin–lattice relaxation time, *T*_1*m*_, related to the recovery of *M_z_*, the z (or longitudinal) *M* component. *M_xy_* extinction and *M_z_* recovery were measured (37 °C) using a Bruker Minispec MQ20, Billerica, MA, USA (static magnetic field B0 = 0.47 T, 20 MHz, Germany), a bench instrument that can be easily hosted in a hospital laboratory. Indeed, it does not need particular safety requirements due to the low magnetic field involved. Each sample was poured into the glass tube (internal diameter 0.008 m), sealed with a proper plastic top just after sample insertion. Then, the glass tube was maintained at 37 °C for about ten minutes before measuring. Finally, it was rapidly inserted into the MQ20 sample holder positioned just above the magnetic field. *M_xy_* extinction (transversal relaxation) was recorded according to the CPMG sequence (Carr–Purcell–Meiboom–Gill [41]) sequence {90° [-τ-180–τ (echo)]_k_ − T_R_} with an 8.36 μs wide 90° pulse, τ = 250 μs and T_R_ (recycle delay) equal to 10 s. *k* represents the number of experimental echoes and it is (approximately) related to the experimental test duration, T_d_, by T_d_ = (2 τ) × k = (2 τ) k’ × (1 + A), where k’ is the number of recorded echoes and A is the number of not recorded echoes. Thus, k = k’ × (1 + A). The trial-and-error procedure adopted to choose k’ (≤1000) and A (≤21) ensured that at the end of the experiment (t = Td), the *M_xy_* intensity (FID or Is(t)) was about 2% of the initial intensity. Consequently, the time interval (T_d_/k’) for data acquisition is equal to 2 τ × (1 + A), and it can differ from sample to sample in reason of the different k’ and A considered to get the desired T_d_. Each relaxation experiment, composed of k’ points, was repeated 36 times (four scans for each of the 9 repetitions performed on the same sample).

*M_z_* recovery (longitudinal relaxation) was recorded according to the saturation recovery sequence consisting of an ensemble of multiple 90-degree RF pulses at relatively short repetition times [42]: {n × [90–Δ/n]-τ-90–Echo acquisition}. n = 10 was considered in this work.

The LF-NMR equipment was validated every day according to an internal procedure named “Daily Check” that makes use of an internal standard. It serves to automatically recalibrate the instrument’s parameters and to check the performance of the system, such as signal strength or temperature stability. The 36 repetitions performed on each sample proved the high reproducibility of the performed measurement (signal intensity). Indeed, the highest values of standard deviation and coefficient of variation were, respectively, 1.77 and 0.038. *T*_2*m*_/*T*_1*m*_ measurements were performed in a blinded fashion.

*T*_2*m*_–*T*_1*m*_ determination, jointly with their discrete and continuous distributions, was performed as detailed in the following sections.

### 4.5. T_2m_ and T_1m_ Determination

*T*_2*m*_ determination was achieved by fitting to the experimental relaxation data the following sum of exponential terms, each one characterized by a different time decay constant (*T*_2*i*_) and weight (*A*_2*i*_) [43]:

(1) $$I(t)\;=\;\sum_{i=1}^{m}A_{2i}e^{\left(-t/T_{2i}\right)}$$

where *I(t)* is the dimensionless signal amplitude that becomes negligible at the end of the relaxation process. The number, *m*, of exponential decays appearing in Equation (1) was determined by a statistical [44] procedure based on the minimization of the product (2 × *m* × χ^2^), where χ^2^ is the sum of the squared errors and 2*m* is the number of model fitting parameters. The average spin–spin relaxation time *T*_2*m*_ can be evaluated by the following equation:

(2) $$T_{2m}=\sum_{i=1}^{m}A_{2i}T_{2i}/\sum_{i=1}^{m}A_{2i}A_{2i\%}=100\;A_{2i}/\sum_{i=1}^{m}A_{2i}$$

A similar approach was followed to determine the average spin–lattice relaxation time (*T*_1*m*_). Indeed, relaxation data have been fitted by the following sum of exponential terms, each one characterized by a different time decay constant (*T*_1*i*_) and weight (*A*_1*i*_) [37]:

(3) $$I(t)\;=\;\sum_{i=1}^{n}A_{1i}\left(1-e^{\left(-t/T_{1i}\right)}\right)$$

where *I*(*t*) is the dimensionless signal amplitude that assumes its maximum value at the end of the relaxation process. The number, *n*, of exponential decays appearing in Equation (3) and the average spin–lattice relaxation time *T*_1*m*_ were determined as in the *T*_2*m*_ case:

(4) $$T_{1m}=\sum_{i=1}^{n}A_{1i}T_{1i}/\sum_{i=1}^{n}A_{1i}A_{1i\%}=100\;A_{1i}/\sum_{i=1}^{\mathrm{mn}}A_{1i}$$

While the *m* couples (*T*_2*i*_, *A*_2*i*_%) and the *n* couples (*T*_1*i*_, *A*_1*i*_%) represent the discrete relaxation time distribution referring to the transversal and longitudinal relaxation, respectively, it is possible to determine the continuous distributions as detailed in the following sections.

### 4.6. Continuous Relaxation Spectrum Determination: Spin–Spin Relaxation (T_2_)

While the *m* couples (*A*_2*i*_ − *T*_2*i*_) represent the spin–spin discrete relaxation spectrum, it is possible to determine the continuous relaxation spectrum according to the Whittal and MacKay approach [45]:

(5) $$I\left(t\right)=\int_{T_{2\min}}^{T_{2\max}}a(T_{2})\exp\left\{-\frac{t}{T_{2}}\right\}dT_{2}$$

where *t* is time, *T*_2_ is the spin–spin or transverse relaxation time and *I*(*t*) is the theoretical intensity. *T*_2*max*_ (=10^4^ ms) and *T*_2*min*_ (=10 ms) indicate, respectively, the lower and upper values that *T*_2_ can assume; *a(T*_2_*)* is the unknown amplitude of the spectral component at the relaxation time *T*_2_, while exp{−t/*T*_2_} represents the decay term. In order to fit the experimental time decay (*Is*(*t*)) by Equation (5), and to get the *T*_2_ distribution (*A*_2*i*_ − *T*_2*i*_), where the unknowns *A*_2*i*_ are given by the product (*ai*(*T*_2*i*_*)* × Δ*T*_2*i*_), the following discretization was applied [39]:

(6) $$I\left(t\right)\approx \sum_{i=1}^{M}a_{i}e^{\left\{-\frac{t}{T_{2i}}\right\}}\left(T_{2i+1}-T_{2i}\right)=\sum_{i=1}^{M}A_{2i}e^{\left\{-\frac{t}{T_{2i}}\right\}}$$

where the range of the *T*_2_ distribution (*T*_2*min*_–*T*_2*max*_) was logarithmically subdivided into *N* = 200 parts (higher *M* values were unnecessary). Ultimately, the adoption of Equation (6) implies the iterative determination of the *M* unknowns *A*_2*i*_, usually a heavy computational task. In order to speed up the iterative procedure, relying on our previous work [46,47,48,49,50] (see also Figure S5), we assume that the unknown *A*_2*i*_ distribution can be safely described by a sum of *M_W_* Weibull equations so that Equation (6) becomes:

(7) $$A_{2i}=\sum_{j=1}^{j=M_{w}}B_{j}(\frac{2\delta_{j}}{\eta_{j}}){(2\frac{T_{2i}-T_{2\min\text{-}j}}{\eta_{j}})}^{\delta_{j}-1}\mathrm{EXP}(-{(2\frac{T_{2i}-T_{2\min\text{-}j}}{\eta_{j}})}^{\delta_{j}})$$

(8) $$I\left(t\right)=\sum_{i=1}^{M}\left[A_{2i}\right]e^{\left\{-\frac{t}{T_{2i}}\right\}}$$

where *Bj*, *δj*, *ηj* and *T*_2*min*-*j*_ are the four fitting parameters competing to each one of the *M_W_* Weibull distributions considered. In so doing, the fitting parameters turn out to be 4× *M_W_*, a much smaller unknown number with respect to the 200 considered in Equation (6). Because of the noise disturbing the measure of *Is*, the fitting procedure must not minimize the χ^2^ statistic, but a smoothed definition of it $\chi_{s}^{2}$:

(9) $$\chi_{s}^{2}=\sum_{i=1}^{M}{\left(\frac{I_{s}\left(t_{i}\right)-I\left(t_{i}\right)}{\sigma_{i}}\right)}^{2}\;+\mu \sum_{i=1}^{M-2}{\left|A_{i+2}-{2A}_{i+1}+A_{i}\right|}^{2}$$

where *σ_i_* is the *i*th datum standard deviation, μ is the weight of the smoothing term (second summation in Equation (9)) proposed by Provencher [51]. Although different criteria can be used to determine μ, the strategy proposed by Wang [52] was applied. Based on this strategy, the correct μ value is that occurring just below the heel (slope variation) of the function ln(χ) vs. ln(μ). In this work, μ = 150 was determined.

### 4.7. Continuous Relaxation Spectrum Determination: Spin–Lattice Relaxation (T_1_)

The strategy adopted to determine the continuous spin–lattice relaxation spectrum is equal to that referring to the spin–spin one. Thus, assuming that the unknown *A*_1*i*_ distribution can be safely described by a sum of *N_W_* Weibull equations, the theoretical intensity can be expressed by

(10) $$A_{1i}=\sum_{j=1}^{j=N_{w}}B_{j}(\frac{2\delta_{j}}{\eta_{j}}){(2\frac{T_{1i}-T_{1\min\text{-}j}}{\eta_{j}})}^{\delta_{j}-1}\mathrm{EXP}(-{(2\frac{T_{1i}-T_{1\min\text{-}j}}{\eta_{j}})}^{\delta_{j}})$$

(11) $$I\left(t\right)=\sum_{i=1}^{N}\left[A_{1i}\right]\left(1-e^{\left\{-\frac{t}{T_{1i}}\right\}}\right)$$

where Bj, δj, ηj and *T*_1*min*-*j*_ are the four fitting parameters competing to each one of the *N* Weibull distributions considered. Due to the noise disturbing the measurement of the experimental time decay (*Is*(*t*)), data fitting was performed by minimizing $\chi_{s}^{2}$ (Equation (9)) instead of χ^2^. Also in this case, the smoothing term μ turned out to be 150.

### 4.8. T_1_–T_2_ Plane

The strategy adopted to fit the relaxation data (i.e., Equations (8) and (11)) allowed to separately determine *T*_1_ and *T*_2_ distributions, and to obtain a 2D representation in the *T*_1_–*T*_2_ plane. Although the determination of the real *T*_1_–*T*_2_ surface would require the simultaneous measurement of *T*_1_ and *T*_2_ distributions according to a proper methodology [53], with the specific aim of proposing a simple and impressive way to simultaneously show the entire *T*_1_ and *T*_2_ distributions, we adopted a simple shortcut consisting in depicting the product of the *T*_1_ and *T*_2_ intensity (*I*_2*Di*_(*T*_2*i*_, *T*_1*i*_)) that can be determined as the product of Equations (8) and (11):

(12) $$I_{2\mathrm{Di}}=A_{2i}\left(T_{2i}\right)\times A_{1i}\left(T_{1i}\right)$$

where *A*_2i_ and *A*_1i_ represent the signal intensities reported in Equations (8) and (11). For clarity of presentation, *I*_2*Di*_ has been normalized by its maximum value in the (*T*_2*i*_, *T*_1*i*_) range considered and multiplied by 100. Notably, provided that *T*_1_ and *T*_2_ distributions are similar [53] (we found a significant statistical correlation among the continuous *T*_1_ and *T*_2_ distribution intensities of each sample), our *T*_1_–*T*_2_ should not be so far from the real *T*_1_–*T*_2_ surface.

### 4.9. Statistics and Statistical Indices

The huge amount of data collected required the definition/use of different indices to allow data comparison for correlation definition.

Microbiome’s data were examined using classical indices such as the Shannon (*Sh*) and Equitability (*Eq*) indices [54]:

(13) $$S_{h}\;=\;-\sum_{j=1}^{s}p_{j}\ln{(p}_{j})$$

(14) $$E_{q}=S_{h}/\ln(s)$$

where *p_j_* represents the % of the entire bacterial community belonging to ASVs, so that Σ*p_j_* = 100, while *s* is the number of ASVs constituting the microbiome.

In addition to the classical indices used to describe the diversity of ASVs in a community (*Sh*, *Eq*), we defined three other indices. The leading idea relies on the definition of the “distance” *d_j_* between the *j*th fraction (*p_j_*) and the average fraction values (p_m_):

(15) dj=pj−pm; pm=∑j=1spjs

Accordingly, each component of the community can be identified by a couple of numbers, (*d_j_*, *p_j_*), that allows it to be collocated in a defined point of the plane (*d_j_* vs. *p_j_*). Finally, it is possible to define the distance, *d_Mj_*, of each component with respect to the average component whose coordinates are (0; *p_m_*)

(16) $$d_{\mathrm{Mj}}=\sqrt[{2}]{{\left(p_{j}-p_{m}\right)}^{2}+d_{j}^{2}}=\sqrt[{2}]{2}\left|d_{j}\right|$$

Relying on *d_Mj_*, it is possible to define the community average value, *d_M_*, compared to the community and express how closely (small *d_M_*) or how widely (big *d_M_*) the community distributes around the mean:

(17) $$d_{M}=\frac{\sum_{j=1}^{s}{p_{j}d}_{\mathrm{Mj}}}{\sum_{j=1}^{s}p_{j}}$$

Notably, the verified existence of a strong inverse correlation between d_M_ and both S_h_ and E_q_ when analyzing microbiome at the phylum and species level (phylum: r_sp_(S_h_ − d_M_) = −0.91, *p* < 10^−4^; r_sp_(E_q_ − d_M_) = −0.94, *p* < 10^−4^; species: r_sp_(S_h_ − d_M_) = −0.95, *p* < 10^−4^; r_sp_(E_q_ − d_M_) = −0.94, *p* < 10^−4^ supports the theoretical robustness of this new index. Anyway, the real advantage of d_M_ over the classical S_h_ and E_q_ indices lies in the possibility of providing a very simple and impressive way for the representation of the microbiome distribution, as depicted in Figure S1.

Relying on the strategy adopted to define d_M_, we can define the other two indices, the first one, $P_{MAX}^{\%}$, representing the maximum percentage *p_j_*:

(18) $$P_{\mathrm{MAX}}^{\%}=\max(p_{j})$$

and the second one, *D*_0_, representing the community distance from an ideal community characterized by *d_M_* = 0 and $P_{MAX}^{\%}$= 0:

(19) $$D_{0}=\sqrt[{2}]{d_{M}^{2}+{\left(P_{MAX}^{\%}\right)}^{2}}$$

The strong correlation between d_M_ and $P_{MAX}^{\%}$, when analyzing microbiome at the phylum and species level (phylum: r_sp_ = −0.97, *p* < 10^−4^; species: r_sp_ = −0.96, *p* < 10^−4^), underlines the strict connection between d_M_ and $P_{MAX}^{\%}$. Consequently, *D*_0_ can be considered a surrogate of d_M._ The *t*-Student test was considered to compare means among healthy and MOLD samples.

The nature of the experimental data distribution (Gaussian or not) was evaluated by the Kolmogorov–Smirnov test (KS-test). Based on KS-test results, the Spearman (r_sp_) or Pearson correlation coefficient (r_p_) was considered to verify possible linear correlations among all parameters. *p* values below 0.05 were considered statistically significant. Statistical analysis was performed by means of the GraphPad InStat.

### 4.10. Disease Index I_D_

In order to easily and synthetically determine the clinical conditions of patients, we defined a “disease index”, *I_D_*, representing the distance between each patient and the average healthy subject (normal condition). *I_D_* evaluation relies on the markers chosen in this work to identify the clinical conditions (*FEV*_1_, TNFα, IL-6 and bacteria concentration, *C_b_*) and on the Euclidean definition of distance:

(20) $$I_{D}=\sqrt[{2}]{{\left(\frac{\mathrm{TNF}\alpha_{P}-\mathrm{TNF}\alpha_{\mathrm{AH}}}{{\mathrm{TNFa}}_{\mathrm{AH}}}\right)}^{2}+{\left(\frac{{IL6}_{P}-{IL6}_{\mathrm{AH}}}{{IL6}_{\mathrm{AH}}}\right)}^{2}+{\left(\frac{C_{b}^{P}-C_{b}^{\mathrm{AH}}}{C_{b}^{\mathrm{AH}}}\right)}^{2}{+(\frac{{\mathrm{FEV}}_{1}^{P}-{\mathrm{FEV}}_{1}^{\mathrm{AH}}}{{\mathrm{FEV}}_{1}^{\mathrm{AH}}})}^{2}}$$

Equation (20), where TNFα_P_, IL6_P_, $C_{b}^{P}$ and ${FEV}_{1}^{P}$ represent the marker values referring to the specific patient, while TNFα_AH_, IL6_AH_, $C_{b}^{AH}$ and ${FEV}_{1}^{AH}$ refer to the marker values averaged on all the healthy subjects considered in this work. In order to equally consider the effect of each marker on *I_D_*, the detachments from normality (represented by TNFα_AH_, IL6_AH_, $C_{b}^{AH}$ and ${FEV}_{1}^{AH}$) have been divided by the respective average value compared to healthy subjects. Clearly, the contribution of FEV_1_ is set to zero when ${\mathrm{FEV}}_{1}^{P}>{\mathrm{FEV}}_{1}^{\mathrm{AH}}$. Similarly, the contribution of the other biomarkers (TNFα_p_, IL6 and C_b_) is set to zero when they are below their respective threshold (TNFα_AH_, ${IL6}_{\mathrm{AH}}$ and $C_{b}^{\mathrm{AH}}$). The definition of *I_D_* makes this index very ductile, as it could also account for other markers that clinicians could consider important. In addition, the relative contribution of each marker could be properly weighed (weight spanning from 0 to 1) according to the clinical importance attributed to each marker. Notably, in case of missing information, the reference values can be retrieved in specialized literature. Finally, it is important to underline that *I_D_* is zero for the average healthy subject and it progressively increases with the worsening of the patient’s clinical conditions.

It is important to remember that for some samples (see Table 1a), FEV_1_ is not available. Thus, for these samples, I_D_ has been evaluated without considering the contribution of FEV_1_. This is not a serious drawback, as, for numerical reasons, the effect of FEV_1_ is not so important. Clearly, this would not take place when introducing a consistent weight to the FEV_1_ contribution.

Finally, Table 6 reports the values measured for each healthy subject.

## Figures and Tables

**Table 1:** (**a**) Patient’s characteristics and clinical conditions. The table reports patient code, sex, age, and clinical information, including pathology (COPD = chronic obstructive pulmonary disease; NCFB = non-cystic fibrosis bronchiectasis), smoking history (1 = current smoker; 2 = non-smoker; 3 = ex-smoker), clinical status (1 = exacerbation; 2 = stable), and *FEV*_1_ (%), when available. (**b**) Characteristics of health controls. The table reports healthy control code, sex, age, smoking history (1 = current smoker; 2 = non-smoker) and *FEV*_1_ (%).

(**a**)						
**Code**	**Sex**	**Age**	**Pathology** **(1—COPD/2—NCFB)**	**Smoked History** **(1—Yes/2—No/3—Ex-Smoker)**	**Clinical Status** **(1—Exacerbation/2—Stable)**	***FEV*** **_1_** **%**
PN1	M	72	1	3	2	39%
PN2	F	75	2	3	1	98%
PN3	F	75	2	3	1	//
PN4	M	73	1	1	1	//
PN5	F	52	2	2	1	//
PN6	M	75	1	1	1	43%
PN7	F	61	2	3	2	67%
PN8	F	69	1	1	2	//
PN9	M	67	2	2	2	//
PN10	F	81	1	1	1	//
PN11	M	68	1	1	1	31%
PN12	M	75	1	1	1	//
PN13	M	72	1	1	1	//
PN14	F	70	1	1	2	64%
PN15	M	67	1	1	2	74%
PN16	M	82	1	3	1	66%
PN17	F	32	2	2	1	16%
PN18	M	65	1	1	1	//
PN19	M	84	1	3	2	78%
PN20	F	76	2	2	2	72%
PN21	M	66	2	3	2	55%
PN22	M	78	1	3	2	74%
PN23	F	57	2	4	1	//
PN24	F	70	1	1	1	//
PN25	M	78	1	3	2	76%
PN26	M	82	1	3	1	34%
PN27	M	76	1	1	1	//
PN28	M	68	1	1	1	44%
PN29	F	71	1	1	2	//
PN30	M	93	1	3	1	100%
PN31	F	58	2	3	2	100%
PN32	F	65	2	2	2	102%
PN33	F	69	1	1	1	52%
PN34	F	77	1, 2	3	1	80%
PN35	M	47	1	1	2	124%
PN36	M	87	1	3	2	82%
PN37	M	74	1	1	1	31%
PN38	M	78	1	1	1	46%
(**b**)						
**Code**			**Sex**	**Age**	**Smoking history**	***FEV*_1_ (%) **
C1			M	62	2	98
C2			M	78	2	81
C3			M	85	2	70
C4			M	64	2	85
C5			F	70	2	87
C6			F	70	2	84
C7			M	58	2	100
C8			M	62	2	94
C9			F	62	2	95
C10			F	76	2	82
C11			M	82	2	80
C12			F	82	2	77
C13			M	63	2	93
C14			M	88	2	70
C15			F	60	2	95
C16			F	59	2	100

**Table 2:** Statistical correlation among the parameters considered (*FEV*_1_, TNFα, IL-6, *C_b_*, *T*_2*m*_). Bold numbers indicate statistically significant correlation (*p* < 0.05) according to the Spearman correlation coefficient r_sp_. “0” indicates no correlation.

r_sp_ *p* < 0.05	*FEV*_1_	TNFα	IL-6	*C_b_*	*T*_2*m*_
*FEV*_1_	1	0	0	0	0
TNFα	0	1	0.35	0.36	−0.33
IL-6	0	0.35	1	0	0
*C_b_*	0	0.36	0	1	−0.5
*T*_2*m*_	0	−0.33	0	−0.5	1

*FEV*_1_: forced expired volume in the first second; TNFα: tumor necrosis factor α; IL-6: interleukin-6; *C_b_*: bacteria concentration; *T_2m_*: spin–spin relaxation time.

**Table 3:** Spearman correlation coefficient (r_sp_) referring to the correlation among statistical indices evaluated at bacterial ASVs level and MOLD parameters (*FEV*_1_, TNFα, IL-6, *C_b_*, *T*_2*m*_.). “0” indicates no correlation.

r_sp_ *p* < 0.05	*FEV*_1_ %	TNFα (pg/mL)	IL-6 (pg/mL)	*C_b_* (ng/μL)	*T*_2*m*_ (ms)
*d_M_* (−)	0	0	0.383	0.34	0
$P_{MAX}^{\%}$ (−)	0	0	0.354	0.35	0
*D*_0_ (−)	0	0	0.367	0.378	0
*Eq* (−)	0	0	−0.326	−0.38	0
*Sh* (−)	0	0	−0.3	−0.44	0.423

*FEV*_1_: forced expired volume in the first second; TNFα: tumor necrosis factor α; IL-6: interleukin-6; *C_b_*: bacteria concentration; *T*_2*m*_: spin–spin relaxation time; *d*_M_: community broadness; $P_{MAX}^{\%}$: maximum percentage *p*_j_ *D*_0_*:* community distance from an ideal community. *Eq*: Equitability index; *Sh*: Shannon index.

**Table 4:** Significant correlations (*p* < 0.05) among the parameters *T*_2*m*_ and *Sh* and the concentration of bacteria genera. *C_b_*_%_ indicates the average percentage of each bacterial genus evaluated in our samples; 16.7% of the selected bacterial genera correlate with *T*_2*m*_, while 21.4% correlate with *Sh*.

	T_2m_	Sh
significant correlations	16.7%	21.4%
genus	Streptococcus	Streptococcus
	(rsp = −0.39; *C_b_*_%_ = 32%)	(rsp = −0.48; *C_b_*_%_ = 32%)
	uncl. Firmicutes	uncl. Firmicutes
	(rsp = −0.43; *C_b_*_%_ = 3.3%)	(rsp = −0.58; *C_b_*_%_ = 3.3%)
	Staphylococcus	Staphylococcus
	(rsp = −0.35; *C_b_*_%_ = 3.1%)	(rsp = −0.42; *C_b_*_%_ = 3.1%)
	uncl. Bacilli	Granulicatella
	(rsp = −0.43; *C_b_*_%_ = 1.5%)	(rsp = −0.37; *C_b_*_%_ = 1.9%)
	Lactobacillus	uncl. Bacilli
	(rsp = −0.43; *C_b_*_%_ = 0.9%)	(rsp = −0.46; *C_b_*_%_ = 1.5%)
	uncl. Streptococcaceae	Lactobacillus
	(rsp = −0.43; *C_b_*_%_ = 0.8%)	(rsp = −0.32; *C_b_*_%_ = 0.9%)
	Corynebacterium	uncl. Streptococcaceae
	(rsp = −0.43; *C_b_*_%_ = 0.2%)	(rsp = −0.49; *C_b_*_%_ = 0.8%)
		uncl. Bacillales
		(rsp = −0.44; *C_b_*_%_ = 0.3%)
		uncl. Coriobacteriaceae
		(rsp = −0.46; *C_b_*_%_ = 0.1%)

*T*_2m_: spin–spin relaxation time; S_h_: Shannon index.

**Table 5:** Existing correlations among the parameters *FEV*_1_, TNFα, IL-6, *C_b_* and *T*_2*m*_, referring to “acute exacerbation” group. r_sp_ is the Spearman correlation coefficient. “0” indicates no correlation.

Acute Exacerbation					
rsp; *p* < 0.05	*FEV*_1_	TNFα	IL-6	*C_b_*	*T*_2*m*_
*FEV*_1_	1	0	0	0	0
TNFα	0	1	0	0.64	−0.62
IL-6	0	0	1	0	0
*C_b_*	0	0.64	0	1	−0.51
*T*_2*m*_	0	−0.62	0	−0.51	1

*FEV*_1_: forced expired volume in the first second; TNFα: tumor necrosis factor α; IL-6: interleukin-6; *C_b_*: bacteria concentration; *T*_2*m*_: spin–spin relaxation time.

**Table 6:** Measured values for the parameters *FEV*_1_, TNFα, IL-6 and *C_b_* for healthy controls.

Control Number	*FEV*_1_ %	TNFα (pg/mL)	IL-6 (pg/mL)	*C_b_* (ng/μL)
C1	98	4.62	10.31	0.27
C2	81	16.16	13.58	0.09
C3	70	21.33	31.75	0.10
C4	85	24.08	107.24	0.55
C5	87	14.9	9.48	0.80
C6	84	17.15	10.98	1.98
C7	100	15.01	2.91	0.20
C8	94	25.29	15.42	1.33
C9	95	5.84	5.25	0.11
C10	82	9.2	4.02	1.33
C11	80	8.04	6.36	0.08
C12	77	12.56	10.37	0.70
C13	93	9.09	22.9	0.70
C14	70	16.79	25.63	0.38
C15	95	11.06	30.02	0.10
C16	100	7.48	10.53	0.13

## Data Availability

The original contributions presented in this study are included in the article/supplementary material. Further inquiries can be directed to the corresponding author(s).

## Supplementary Materials

The following supporting information can be downloaded at: https://www.mdpi.com/article/10.3390/ijms27146355/s1. References [55,56,57,58] are cited in Supplementary Materials.

## Abbreviations

The following abbreviations are used in this manuscript:

ASVs	Amplicon sequence variants
COPD	Chronic obstructive pulmonary disease
NCFB	Non-cystic fibrosis bronchiectasis
MOLD	Muco-obstructive lung disease
FEV_1_	Forced expired volume in the first second
LF-NMR	Low-field nuclear magnetic resonance
T_2m_	Spin–spin relaxation times
T_1m_	Spin–lattice relaxation times
CF	Cystic fibrosis
C_b_	Bacterial concentration
Sh	Shannon index
Eq	Equitability index
I_D_	Disease index
aEX	Acute exacerbation
ST	Stable
