# Supplementary material for: Pilot Study on the Use of Low-Field Nuclear Magnetic Resonance as a Noninvasive Tool for Monitoring Mucus in Obstructive Lung Diseases
Source: Int J Mol Sci. 2026 Jul 17;27(14):6355. doi: 10.3390/ijms27146355 (PMC13409956; doi:10.3390/ijms27146355)
Supplement: Supplementary file 1 [file ijms-27-06355-s001.zip › ijms-4384989-supplementary.pdf]

Pilot Study on the Use of Low-Field Nuclear Magnetic Resonance as a Noninvasive Tool for Monitoring Mucus in Obstructive Lung Diseases

Supplementary information

1) *Microbiome representation*

MOLD worsening reflects in a reduction of bacteria diversity due to the prevalence of a limited number of Amplicon Sequence Variants (ASVs) as depicted in Figure S1 for two representative patients (PN16 and PN20 of Table 1) and a typical healthy sample. Indeed, we can see that the healthy sample is characterized by a high number of bacteria AVSs (N_s = 1224) and a small mean distance ($d_M \approx 3.4$) between the average percentage and that competing to each bacteria AVSs. In addition, the community distance (D_0) from an ideal healthy community characterized by $d_M = 0$ and $P^{\%}_{MAX} = 0$ is small.

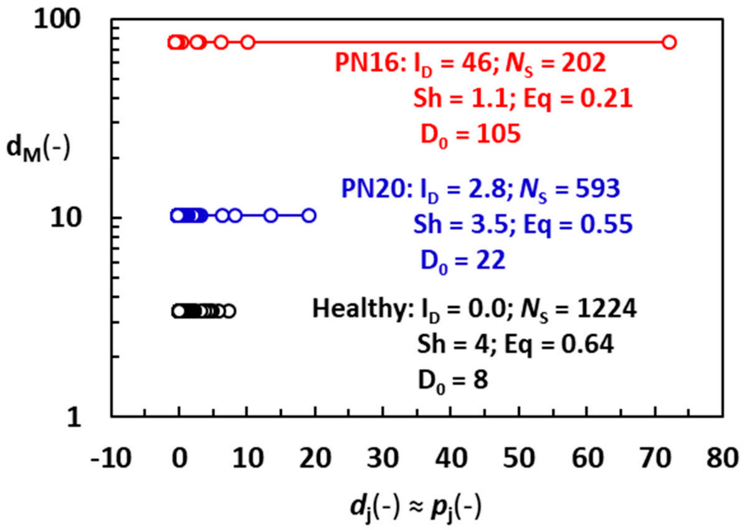

Figure *S1*. Variation of microbiome characteristics with disease progression. Healthy sample is characterized by a vanishing disease index, I_D, and a huge number, N_s, of bacterial AVSs characterized by a similar percentage (small d_M -see eq.(17) and small D_0 –see equation (19)). Disease progression (PN20 and, then, PN16) implies the increase of d_M, D_0 and I_D while N_s reduces. At the same time, the appearance of a dominating bacteria AVSs arises (the last blue and red circles on the right). As the average value of the bacteria AVSs percentage (p_m) is always low (< 1), the distance, d_j, pertaining to each bacteria AVSs, is very close to the percentage of each bacteria AVSs (p_j, see equation (15)).

On the contrary, with MOLD worsening (PN20 and PN16), I_D, d_M and D_0 increase, N_s decreases and one AVSs becomes clearly prevalent, i.e. lung microbiome diversity decreases as also proved by the variations of the *Sh* and the *Eq* indices.

Notably, Figure S1 underlines that the use of the new statistical indices (d_M, $\boldsymbol{P^{\%}_{MAX}}$ and D_0) allows a simple and impressive way to graphically describe the bacteria distribution characteristics of microbiome. In addition, it is interesting to underline the inverse correlation occurring between d_M and the microbiome AVSs number N_s.

2) Benjamini-Hochberg test

This test is aimed at the evaluation of the false positives when conducting multiple comparisons as shown in Table 4**,** where the study of the correlation between T_{2m}/FEV_1/S_h/TNFα and the concentration (C_b) of the most common bacteria genera ($m = 42$) of our sputum samples is reported. According to this test, the correlation scores are ranked according to their "*p*" value, starting from the smallest (rank $k = 1$) to the biggest one (rank $k = m$). Then, the test searches for the highest rank (k_{max}) for which $p_k < (k{*}q)/m$, where p_k is the kth score, k is its rank, m is the total number of tests (42 in our case) and q is the level of the False Discovery Rate (FDR). All the tests characterized by $k \leq k_{max}$ are considered truly positive. In Figure S2 the variation of the truly positive test percentage (vertical axis) versus the FDR level q referring to the Sh (red dots) and T2m (blue dots).

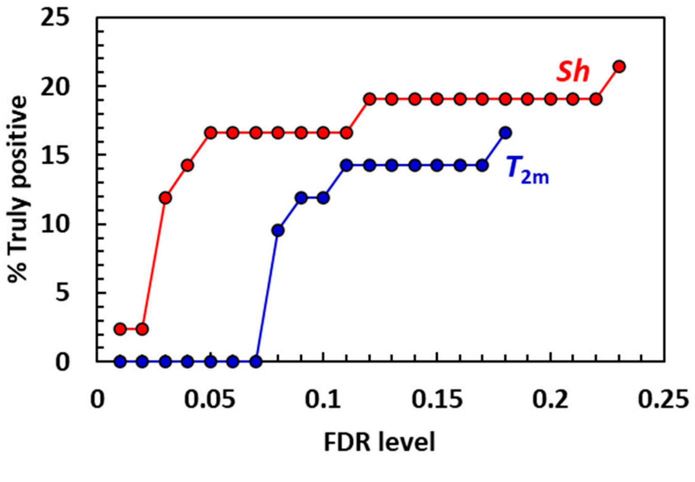

Figure S2

As the score of this test depends on the FDR level (q), we evaluated the percentage of the truly positive tests for different q values as reported in Figure S2 for T_{2m} and S_h, the only parameters

showing a correlation with the concentration of some of the most common bacteria genera found in our sputum samples. While the correlation between S_h and C_b takes place also when the False Discovery Rate (FDR) level q is equal to 0.01 (see Figure S2), for T_{2m} the percentage of correlation is bigger than zero only when FDR level > 0.07. For $q \geq 0.18$, the truly positive percentage is equal to 16.7%.

3) Table S1.

Among all the biomarkers considered in this work, only *IL- 6* correlates with the indices describing the microbiome distribution of aEX patients. r_{sp} is the Spearman correlation coefficient.

Acute exacerbation					
r_{sp}	$\boldsymbol{d_M}$	$\boldsymbol{P^{\%}_{MAX}}$	$\boldsymbol{D_0}$	$\boldsymbol{E_q}$	$\boldsymbol{S_h}$
IL-6	0.57	0.515	0.53	-0.5	-0.48
p =	0.0056	0.014	0.0109	0.0167	0.0214

4) *MICROBIOMA in MOLD clustered in aEX and ST sample*

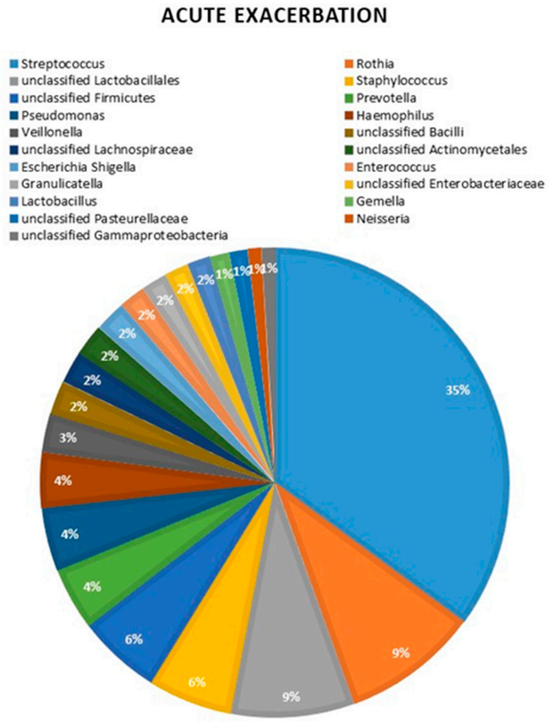

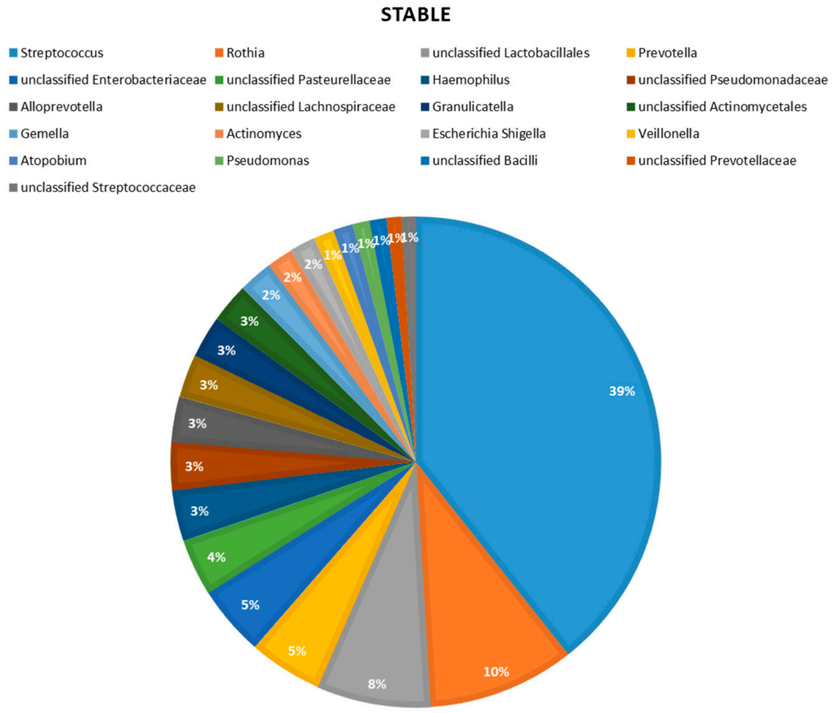

Figure S3

aEX (Acute exacerbation, Figure S3, microbiome composition at the genus level) group shows a significant presence of *Lactobacilli, Staphylococcus* and *Pseudomonas*, genera frequently associated with lung infection, while stable group is characterized by the presence of *Prevotella* (similarly to healthy group), *Alloprevotella*, *unclassified Enterobacteriaceae* and *Pasteurellaceae*. *Streptococcus, Rothia, Haemophilus* and *Veillonella* are present with significant frequency in both aEX and ST (Stable) groups. Interestingly, T_{2m} correlation with *Lactobacilli* (r_{sp} = -0.46) and *Staphylococcus* (r_{sp} = -0.43) occurs in the aEX group, while T_{2m} correlates with *Streptococcus* (r_{sp} = -0.57) in the ST group.

4) Factors affecting T_{2m} and T_{1m}

It is well known that NMR relaxation times (T_{2m} and T_{1m}) are affected by numerous factors including water mobility, viscosity, chemical exchange, macromolecular interactions, susceptibility effects, temperature, magnetic field intensity, solid content and sample micro/nano-structure.

For what concerns the effect of **solid content**, we verified [2] the T_{2m} decrease with the increase of the concentration of alginates, mucins, DNA and albumin (the most important solid components in pathological sputum) in an artificial sputum (aqueous medium). Of course, the entity of T_{2m} reduction with the increase of solid concentration depends on the specific solid. As a matter of fact, considering the typical alginates, mucins, DNA and albumin concentration in pathological sputum, T_{2m} value of our artificial sputum was very close to that of a real CF sample (about 300 ms). In addition, in a recent master thesis [55] we experimentally proved the inverse correlation between T_{2m} and the solid content in real CF and COPD sputum.

The relation between T_{2m} and **nanostructure** relies on the seminal works by Scherer and Chui [28], who theoretically established the connection between the relaxation time (T_{1m} and T_{2m}) and the system nanostructure for gel-like systems. Indeed, system nanostructure is strictly connected to the ratio between solid surface (S) available for interaction with the liquid molecules pervading the system and occupying a volume V. As S/V increases with solid content and it depends on nanostructure characteristics (the smaller the mesh size, the higher the S/V ratio), the dependence of T_{2m} and T_{1m} on solid content and nanostructure is straightforward. Relaying on T_{2m}-T_{1m} dependence on S/V, [56] could follow the thermal gelation of Chitosan/β-Glycerophosphate solutions. Moreover, the relation between nanostructure and relaxation times has been deepened in some papers of ours [29,30] focusing on the determination of the end-to-end distribution length (polymeric systems) by means of low field NMR. This allowed to implement the original Flory theory that assumes a gaussian end-to-end distribution length. Finally, it is important to underline that the relation between T_{2m}-T_{1m} and the mesh size (one of the most important characteristics of gel-like systems) strictly holds only in the case of fast diffusion (fast exchange limit) when liquid molecules (water) is high in comparison to the thickness of the so called "bound" water, i.e. the thin layer of water molecules close to the solid surface (polymer chain surface) whose relaxation time is highly affected by the solid surface [43]. Fortunately, in gel-like systems, this condition is usually met [46].

As **viscosity** increases with polymer concentration and depends on system nanostructure [57] it is reasonable that it inversely correlates with T_{2m} and T_{1m}. Indeed, for example, Abrami et al. [24] found an inverse correlation between T_{2m} and the zero shear viscosity (as viscosity depends on the shear rate, this paper focusses the attention on vanishing shear rate) of cystic fibrosis sputa.

Temperature and **magnetic field intensity** represent other important factors affecting T_{2m} and T_{1m}. In particular, while temperature increase reflects in longer T_{2m} and T_{1m}, magnetic field increase implies T_{1m} increase but does not determine a significant T_{2m} variation. This is the reason why LF-NMR must be performed at the same temperature and magnetic field intensity in order to make possible proper comparisons among different samples.

Clearly also the **susceptibility** of all the sample components is a fundamental aspect for the T_{2m} and T_{1m} determination. For example, the presence of ferromagnetic components makes very difficult, if not impossible, getting structural information by means of LF-NMR as it happens in the case of liver that is rich in iron. Indeed, iron presence is the main reason for T_{2m}-T_{1m} decrease.

Finally, it is important to underline that, traditionally, the determination of the longitudinal relaxation time T_{1m} was preferred to the determination of the transversal relaxation time T_{2m} for reasons connected to **water mobility**. However, as detailed in [58], these aspects no longer represent a serious problem for the T_{2m} determination.

5) *Considerations on the determination of the continuous T_2 and T_1 distributions*

Basically, the idea of using one or more Weibull equations to get the time distributions (T_2 or T_1) was born many years ago [47] when we realized that all the time distributions we got by means of the classical Provencher approach (on which CONTIN relies) [45,51] could be properly fitted by one or more of Weibull equations. In all the following papers of ours [46, 48, 49, 50] we could verify that the results coming from the Provencher (CONTIN) approach were substantially coincident with those of our approach. We were not so surprised by this result in the light of universality character of Weibullian equations that can properly describe many different physical phenomena such as fluctuations in turbulent flows or plasmas, magnetic O(2) models, Bose gases, self- similar processes, 1/fα noise, liquid crystals, and glassy matrices [30].

In order to support the reliability of our approach, Figure S4 and Figure S5 show the comparison among our outcomes and those of the classical Provencher (CONTIN) approach in the case of patients PN20 and PN27, i.e. the patients considered in Figure 3 of the main text.

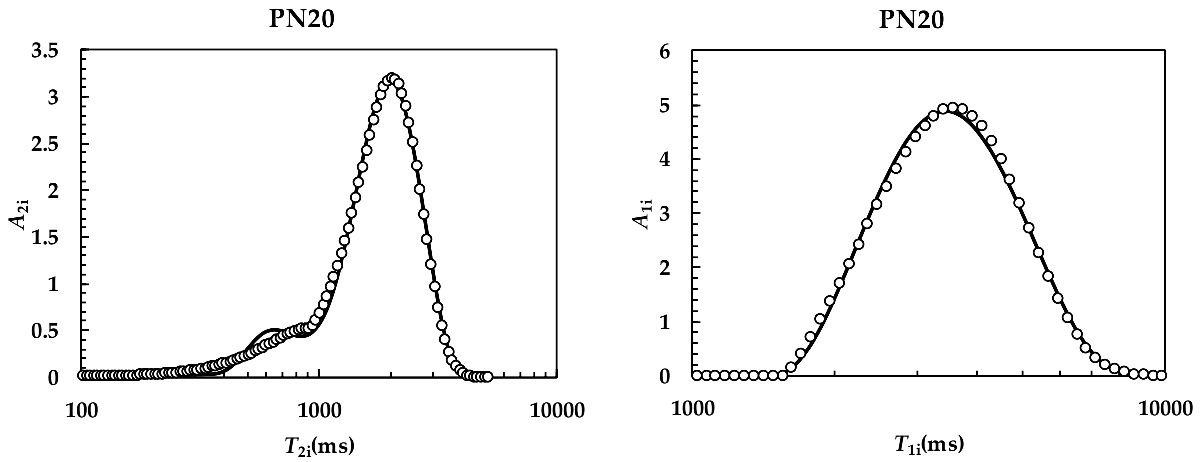

Figure S4

In Figure S4 we show for patient PN20, the comparison between the T_2 (left) and the T_1 (right) distributions coming from our approach (Weibull, symbols) and that by Provencher-CONTIN (solid line). The average relaxation times are: $T_{2\text{m-weib}}$ = 1803.6 ms, $T_{2\text{m-weib}}$ = 1802.4 ms; $T_{1\text{m-weib}}$ = 3560.8 ms, $T_{1\text{m-weib}}$ = 3557.1 ms.

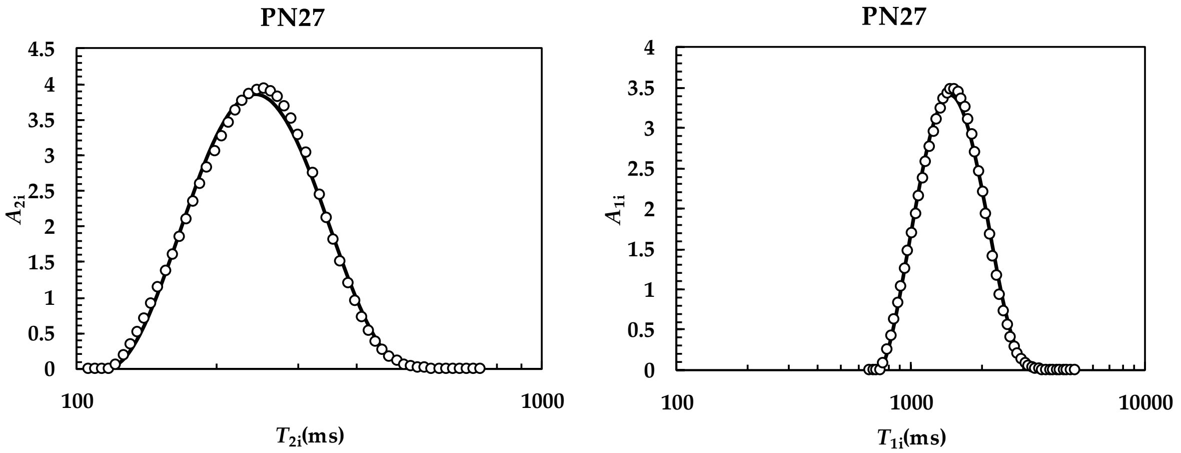

Figure S5

In Figure S5 we show for patient PN27 the comparison between the T_2 (left) and the T_1 (right) distributions coming from our approach (Weiubull, symbols) and that by Provencher-CONTIN (solid line). The average relaxation times are: $T_{2\text{m-weib}}$ = 266.4 ms, $T_{2\text{m-weib}}$ = 265.9 ms; $T_{1\text{m-weib}}$ = 1447.4 ms, $T_{1\text{m-weib}}$ = 1445.5 ms.
